# A novel multi-class imbalanced EEG signals classification based on the adaptive synthetic sampling (ADASYN) approach

**DOI:** 10.7717/peerj-cs.523

**Published:** 2021-05-14

**Authors:** Adi Alhudhaif

**Affiliations:** Department of Computer Science, College of Computer Engineering and Sciences in Al-kharj, Prince Sattam bin Abdulaziz University, Al-Kharj, Saudi Arabia

**Keywords:** Multi-class classification, Random forest, Adaptive synthetic (ADASYN) sampling approach, EEG signals

## Abstract

**Background:**

Brain signals (EEG—Electroencephalography) are a gold standard frequently used in epilepsy prediction. It is crucial to predict epilepsy, which is common in the community. Early diagnosis is essential to reduce the treatment process of the disease and to keep the process healthier.

**Methods:**

In this study, a five-classes dataset was used: EEG signals from different individuals, healthy EEG signals from tumor document, EEG signal with epilepsy, EEG signal with eyes closed, and EEG signal with eyes open. Four different methods have been proposed to classify five classes of EEG signals. In the first approach, the EEG signal was first divided into four different bands (beta, alpha, theta, and delta), and then 25 time-domain features were extracted from each band, and the main EEG signal and these extracted features were combined to obtain 125-time domain features (feature extraction). Using the Random Forests classifier, EEG activities were classified into five classes. In the second approach, each One-Against-One (OVO) approach with 125 attributes was split into ten parts, pairwise, and then each piece was classified with the Random Forests classifier. The majority voting scheme was used to combine decisions from the ten classifiers. In the third proposed method, each One-Against-All (OVA) approach with 125 attributes was divided into five parts, and then each piece was classified with the Random Forests classifier. The majority voting scheme was used to combine decisions from the five classifiers. In the fourth proposed approach, each One-Against-All (OVA) approach with 125 attributes was divided into five parts. Since each piece obtained had an imbalanced data distribution, an adaptive synthetic (ADASYN) sampling approach was used to stabilize each piece. Then, each balanced piece was classified with the Random Forests classifier. To combine the decisions obtanied from each classifier, the majority voting scheme has been used.

**Results:**

The first approach achieved 71.90% classification success in classifying five-class EEG signals. The second approach achieved a classification success of 91.08% in classifying five-class EEG signals. The third method achieved 89% success, while the fourth proposed approach achieved 91.72% success. The results obtained show that the proposed fourth approach (the combination of the ADASYN sampling approach and Random Forest Classifier) achieved the best success in classifying five class EEG signals. This proposed method could be used in the detection of epilepsy events in the EEG signals.

## Introduction

EEG signals include the recording and analysis of electrical signals produced by the brain. EEG is an essential clinical tool for the imaging and diagnosis of neurological diseases with epilepsy. Epilepsy is characterized by a body movement that results in excessive discharge of groups in brain cells and transition disorders, and sudden changes in mental functions. Epileptic EEG signals from the scalp are characterized by high amplitude and synchronized periodic waveforms (Patnaik & Manyam, 2008; Acir et al., 2005; Ozdemir & Polat, 2020; Daldal, Nour & Polat, 2020; Daldal, Polat & Guo, 2019; Khairullah, Arican & Polat, 2020).

**Figure 1:**
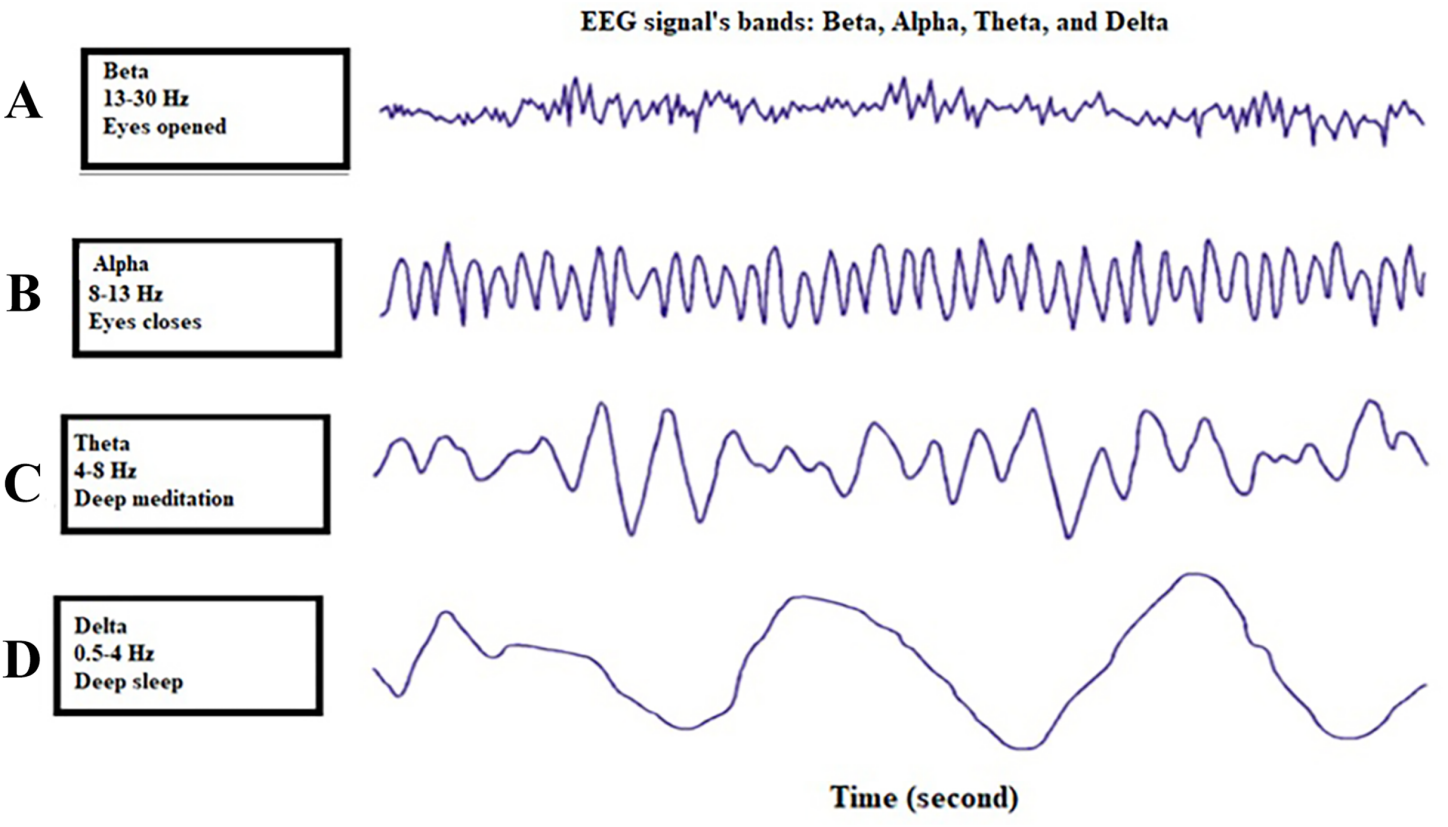
Four different bands of the EEG signal (Vallat, 2018). (A) shows the beta wave in the EEG signals, (B) gives the signal of alpha, (C) denotes the theta signal extracted from the EEG signal, and (D) shows the delta signal component in the EEG signal.

Along with neural activities of the brain, it can produce various signals to be used in multiple areas. EEG signals recorded depending on brain activity are divided into four different classes. These EEG bands are Beta, Alpha, Theta, and Delta. Figure 1 shows the EEG signal bands (Vallat, 2018).

The characteristics and information about the four different bands of the EEG signal are given in Table 1. Table 1 presents the characteristic information belonging to each EEG band (Dingle et al., 1993).

**Table 1:** The characteristics and information about the four different bands of the EEG signal Dingle et al. (1993).

Frequency Band Name	Frequency Bandwidth	The characteristics and information
Raw EEG signal	0–45 Hz	Awake
Delta	0.5–4 Hz	Deep sleep
Theta	4–8 Hz	Drowsy
Alpha	8–13 Hz	Relaxed
Beta	13–30 Hz	Engaged

There are many studies in the literature about the classification of EEG signals. Among them, Patnaik et al. used the Wavelet Transform (WT) and feed-forward backpropagating artificial neural network (ANN) classification for the classification of EEG signals (Patnaik & Manyam, 2008). Chen et al. (2020) proposed a two-phase hybrid method to detect epilepsy status from EEG signals. In the first phase, they extracted attributes from the EEG signals using the autoregressive moving average (ARMA) model and then achieved high classification successes by classifying them with the support vector machine (SVM). Yedurkar & Metkar (2020) proposed a new method for locating the epileptic region and preventing the artifacts that occur in obtaining physiological signals from our body. They proposed a plan called multi-resolution analysis and adaptive filtering (MRAF) and applied it to the diagnosis of EEG epilepsy. In another study, Lian et al. (2020) detected EEG epilepsy by Convolutional Neural Network (CNN) method and compared it with other methods. George et al. (2020) proposed a new hybrid method for classifying EEG signals. This hybrid method consists of two stages: artificial neural network with particle swarm optimization (PSO) and tunable-Q wavelet transform (TQWT). In another study, Ramos-Aguilar et al. (2020) has extracted new attributes from the EEG signals for epileptic seizure detection and applied them to the detection of epilepsy disease.

Apart from the above studies, four new methods have been proposed and compared with each other to classify five classes of EEG signals. All four different methods are a common feature, and feature extraction parts are the same. The difference between methods is the algorithms used and data modeling. Four different methods have been proposed to classify five classes of EEG signals. In the first approach, the EEG signal was first divided into four different bands (beta, alpha, theta, and delta), and then 25 time-domain features were extracted from each band, and the main EEG signal and these extracted features were combined to obtain 125-time domain features (feature extraction). Using the Random Forests (or Bagged Trees) classifier, EEG activities were classified into five classes. In the second approach, each One-Against-One (OVO) approach with 125 attributes was split into ten parts, pairwise, and then each piece was classified with the Random Forests classifier. The majority voting scheme was used to combine decisions from the ten classifiers. In the third proposed method, each One-Against-All (OVA) approach with 125 attributes was divided into five parts, and then each piece was classified with the Random Forests classifier. The majority voting scheme was used to combine decisions from the five classifiers. In the fourth proposed approach, each One-Against-All (OVA) approach with 125 attributes was divided into five parts. Since each piece obtained had an imbalanced data distribution, an adaptive synthetic (ADASYN) sampling approach was used to stabilize each piece. Then, each balanced piece was classified with the Random Forests classifier. The majority voting scheme was used to combine decisions from the five classifiers.

The rest of the article was created as follows. In the second part, material and method parts are given in detail. In the third part, experimental results are given separately. Findings and discussion, which is the last part, are also given in the fourth part.

## Materials & methods

### Multi-class EEG signal dataset

**Figure 2:**
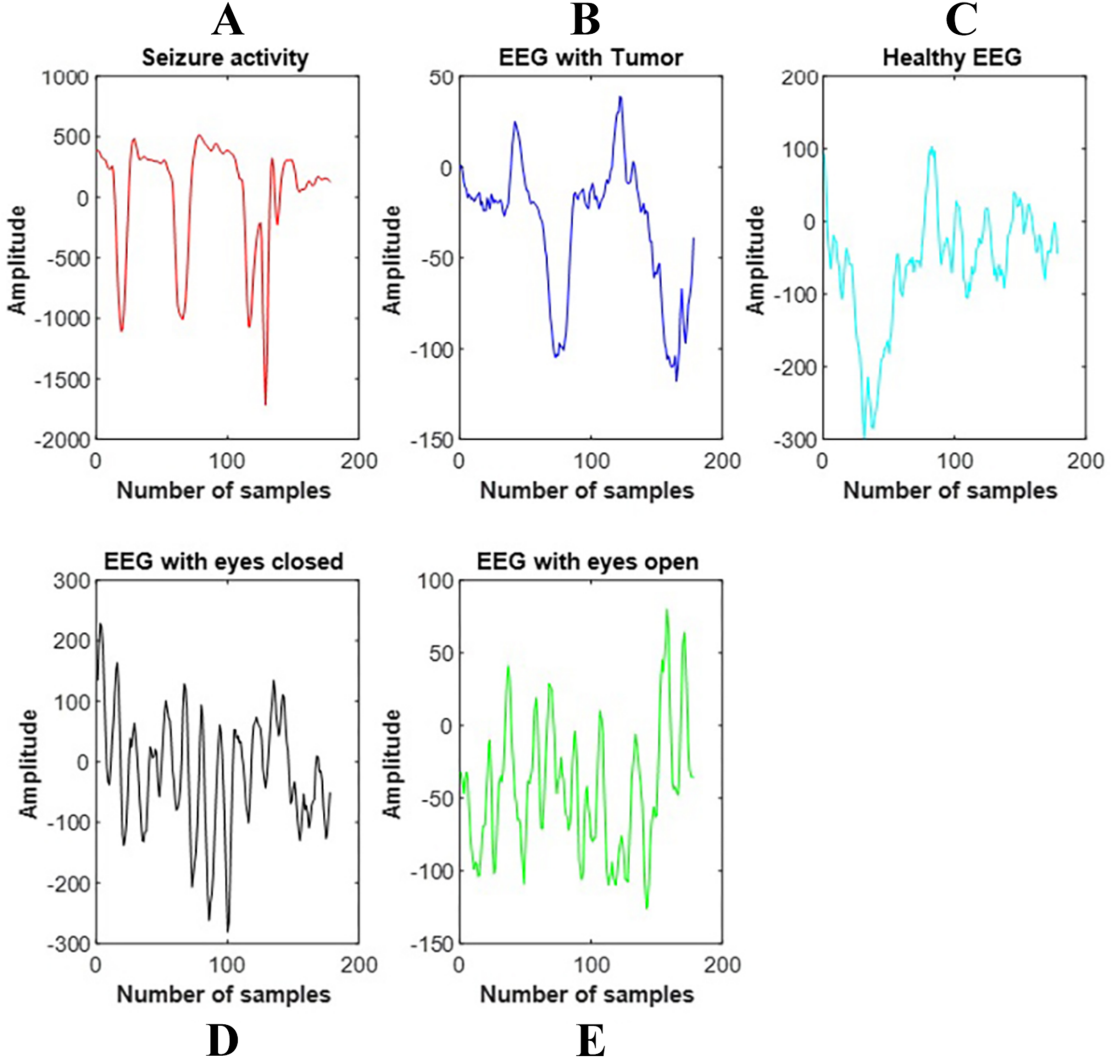
The signals of each band composing the EEG signal. (A) shows the EEG signals having seizure activity, (B) gives the EEG signal with tumor, (C) denotes the healthy EEG signal, (D) shows the EEG signal with the eyes closed, and (E) shows the EEG signal with the eyes open.

The dataset used in the classification of multi-class brain signals was taken from the UCI (the University of California at Irvine) machine learning repository (http://archive.ics.uci.edu/ml/datasets/Epileptic+Seizure+Recognition). The raw EEG signal consists of 4,097 points. The team that created the dataset reduced each EEG signal recording to 178 samples and divided it into one-second epochs to simplify the EEG epilepsy problem. There is a total of five classes in the dataset. These are the EEG signal showing epilepsy, the EEG signal received from the tumor site, the healthy EEG signal, the EEG signal with eyes open, and the EEG signal with eyes closed. Figure 2 shows the signals of each band composing the EEG signal.

### The proposed hybrid methods

In this study, four different approaches were proposed for the classification of multi-class EEG signals. Each approach is given in detail in the sections below.

In the systems given in each block diagram, 25-time domain features were obtained from the EEG signal first. This part is called feature extraction. These extracted 25-time domain features (25-TDF) are given in Table 2 (Polat, 2020; Uçar et al., 2017; Uçar et al., 2018; Wallisch et al., 2014).

**Table 2:** The extracted 25-time domain features from each band of EEG signals in our study.

Number of the feature in EEG signals dataset	Name of the feature	Its equation
1	Kurtosis	$x_{kur}=\frac{\sum_{i=1}^{n}(x(i)-\bar{x}{)}^{4}}{(n-1)S^{4}}$
2	Skewness	$x_{ske}=\frac{\sum_{i=1}^{n}{\left(x_{i}-\bar{x}\right)}^{3}}{(n-1)S^{3}}$
3	*IQR	IQR=iqr(x)
4	DK	$DK=(S/\bar{x})100$
5	Geometric Mean	$G=\sqrt[{n}]{x_{1}+\cdots +x_{n}}$
6	Harmonic Mean	$H=n/(\frac{1}{x_{1}}+\cdots +\frac{1}{x_{n}})$
7	Activity-Hjort Parameters	$A=S^{2}$
8	Mobility-Hjort Parameters	$M=S_{1}^{2}/S^{2}$
9	Complexity-Hjort Parameters	$C=\sqrt{{\left(S_{2}^{2}/S_{1}^{2}\right)}^{2}-{\left(S_{1}^{2}/S^{2}\right)}^{2}}$
10	*Maximum	$x_{max}=max(x_{i})$
11	Median	$\tilde{x}=\{\begin{array}{cc} x_{\frac{n+1}{2}} & :xodd\\ \frac{1}{2}(x_{\frac{n}{2}}+x_{\frac{n}{2}+1}) & :xeven \end{array}$
12	*Mean Absolute Deviation	MAD=mad(x)
13	*Minimum	$x_{min}=min(x_{i})$
14	*Central Moments	CM=moment(x,10)
15	Mean	$\bar{x}=\frac{1}{n}\sum_{i=1}^{n}=\frac{1}{n}(x_{1}+\cdots +x_{n})$
16	Average Curve Length	$CL=\frac{1}{n}\sum_{i=2}^{n}|x_{i}-x_{i-1}|$
17	Average Energy	$E=\frac{1}{n}\sum_{i=1}^{n}x_{i}^{2}$
18	Root Mean Squared	$X_{rms}=\sqrt{\frac{1}{n}\sum_{i=1}^{n}|x_{i}{|}^{2}}$
19	Standard Error	$S_{\bar{x}}=S/\sqrt{n}$
20	Standard Deviation	$S=\sqrt{\frac{1}{n}\sum_{i=1}^{n}\left(x_{i}-\bar{x}\right)}$
21	Shape Factor	$SF=X_{rms}/(\frac{1}{n}\sum_{i=1}^{n}\sqrt{|x_{i}|})$
22	*Singular Value Decomposition	SVD=svd(x)
23	*25% Trimmed Mean	T25=trimmean(x,25)
24	*50% Trimmed Mean	T50=trimmean(x,50)
25	Average Teager Energy	$TE=\frac{1}{n}\sum_{i=3}^{n}\left(x_{i-1}^{2}-x_{i}x_{i-2}\right)$

**Note:**

The feature was computed using MATLAB, IQR Interquartile Range, DK Coefficient of Variation. In formulas, *x* it represents the signal. The properties with “*” were calculated with the help of the MATLAB library.

**Figure 3:**
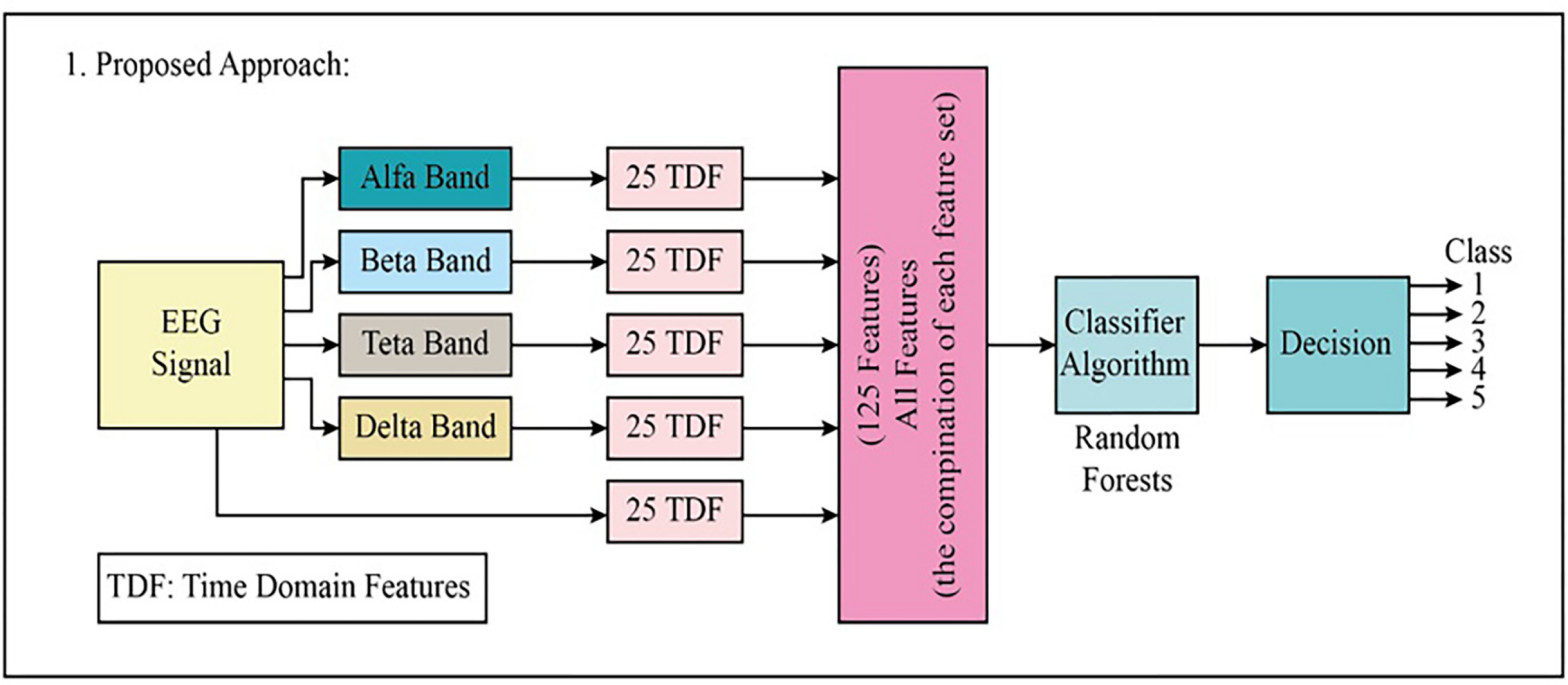
The block diagram of the proposed first approach to classifying the multi-class EEG signals.

In the first proposed method, besides the EEG signal, 25-TDF features were extracted from four different frequency bands extracted from the EEG signal and then combined to create a comprehensive feature set consisting of the 125-TDF features. Then, using the 125-TDF feature set, the EEG signals were classified into five classes using the Random Forests (RF) classifier. The block diagram of the proposed first approach is given in detail in Fig. 3. The class information after decision making is here:

1. Epileptic seizure
2. EEG signals with tumor region
3. Healthy EEG signals
4. EEG signals with eyes closed
5. EEG signals with eyes open

**Figure 4:**
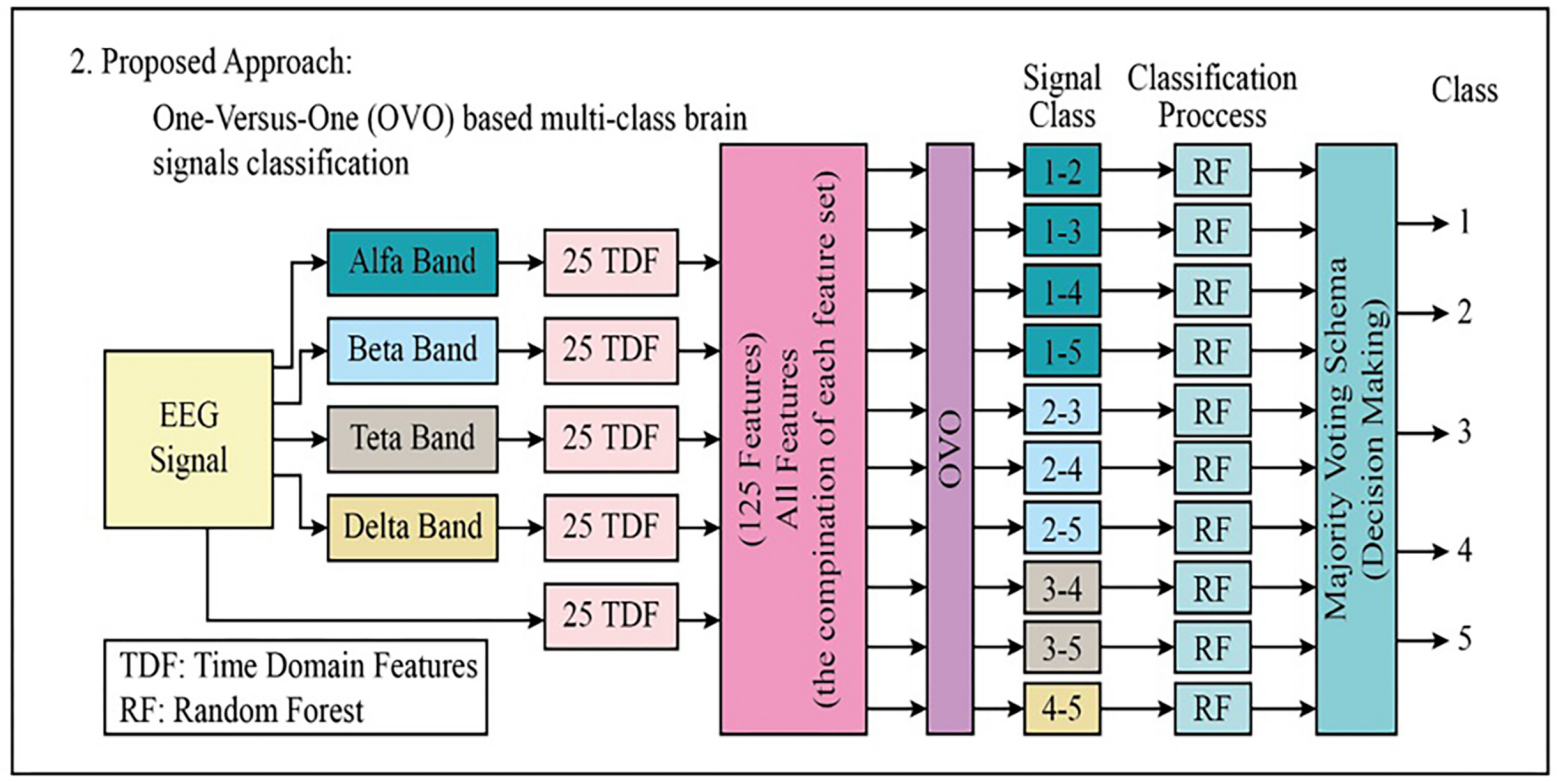
The block diagram of the proposed second approach to classifying the multi-class EEG signals.

In the second proposed approach, the 125-TDF feature set is briefly mentioned as each block schema is common. After the 125-TDF feature set was created, the five-class EEG signals data set was split into ten pieces using one-against-one (OVO). Each piece is classified with Random Forests (RF). The majority voting scheme was used to combine the outputs from each RF classifier (total pieces: 10). The block diagram of the proposed second approach is shown in detail in Fig. 4.

**Figure 5:**
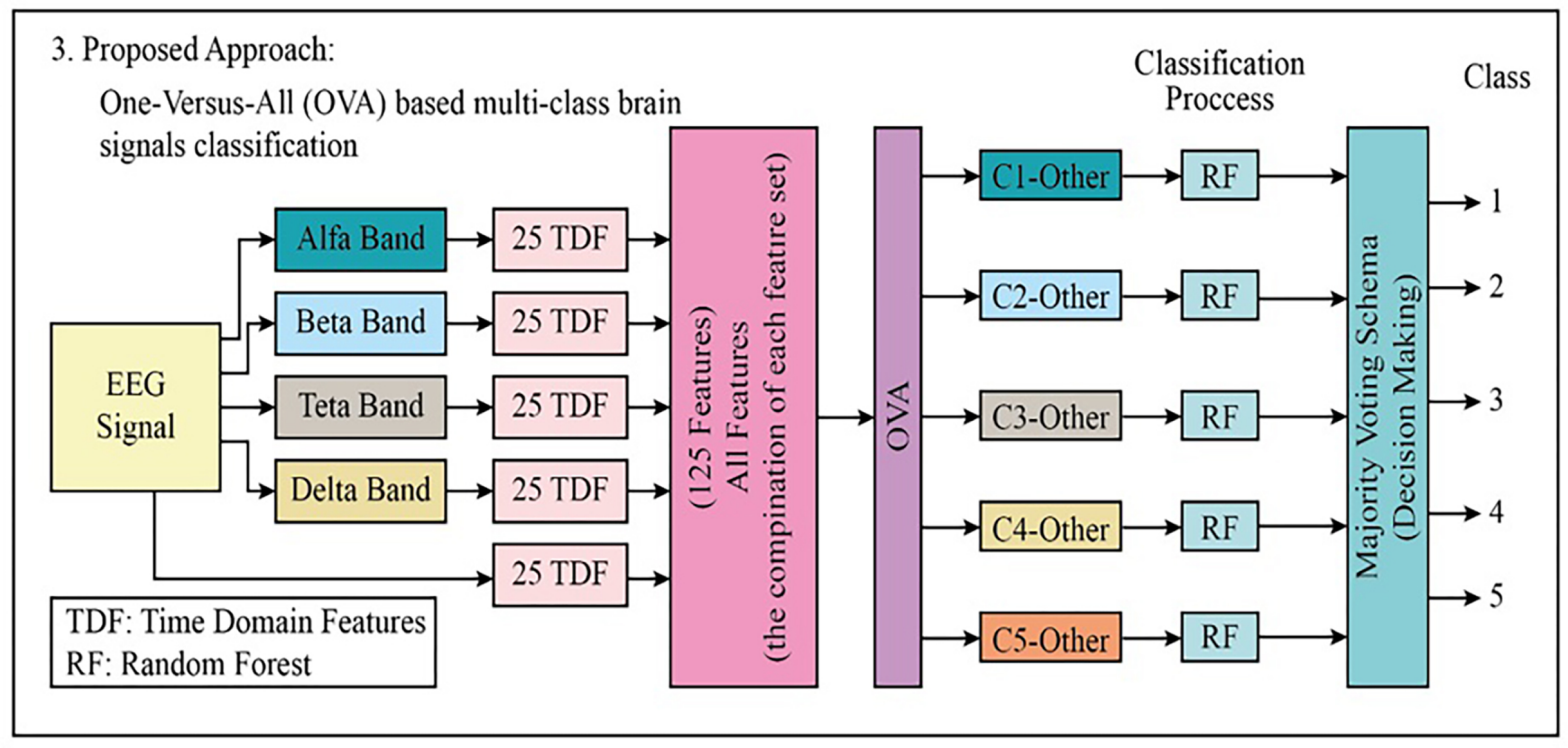
The block diagram of the proposed third approach to classifying the multi-class EEG signals.

As for the third proposed approach, the 125-TDF feature set is briefly mentioned as each block schema is common. After the 125-TDF feature set was created, the five-class EEG signals data set was split into five pieces using one-against-all (OVA). Each piece is classified with Random Forests (RF). The majority voting scheme was used to combine the outputs from each RF classifier (total pieces: 5). Figure 5 depicts the block diagram of the proposed third approach.

**Figure 6:**
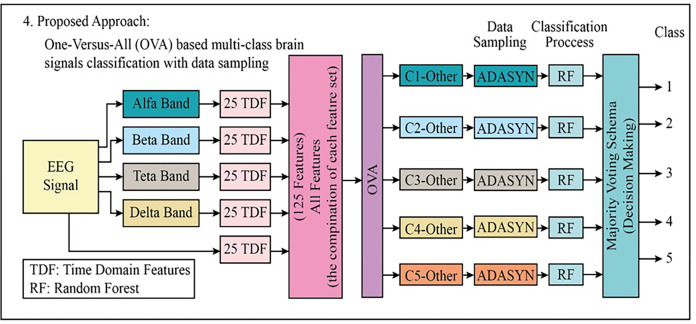
The block diagram of the proposed fourth approach to classifying the multi-class EEG signals.

In the fourth approach, which gives the highest performance in the classification of multi-class EEG signals, the EEG signal and 125 bands in total were extracted from the four bands. Then, with the OVA approach, five different datasets were obtained. Adaptive synthetic (ADASYN) sampling approach (He et al., 2008) has been used to transform the imbalanced datasets to balanced datasets before the classification stage since the data distributions in each piece are imbalanced (class 1-others (the combination of class 2, class 3, class 4, and class 5)). Then, Random Forests (RF) were used to classify five different data sets that became balanced. The majority voting scheme has been used to combine the outputs from each RF classifier (total pieces: 5). Figure 6 explains the block diagram of the proposed fourth approach.

### Adaptive synthetic (ADASYN) sampling approach

Any dataset used for classification is most imbalanced. In the dataset, one group is infrequent (minority class), while in the other set is more data (majority class). In such cases, machine learning algorithms cannot perform well. In the classification of unbalanced data sets, data sampling methods are used to increase the performance of machine learning classification algorithms (https://medium.com/@ruinian/an-introduction-to-adasyn-with-code-1383a5ece7aa).

**Figure 7:**
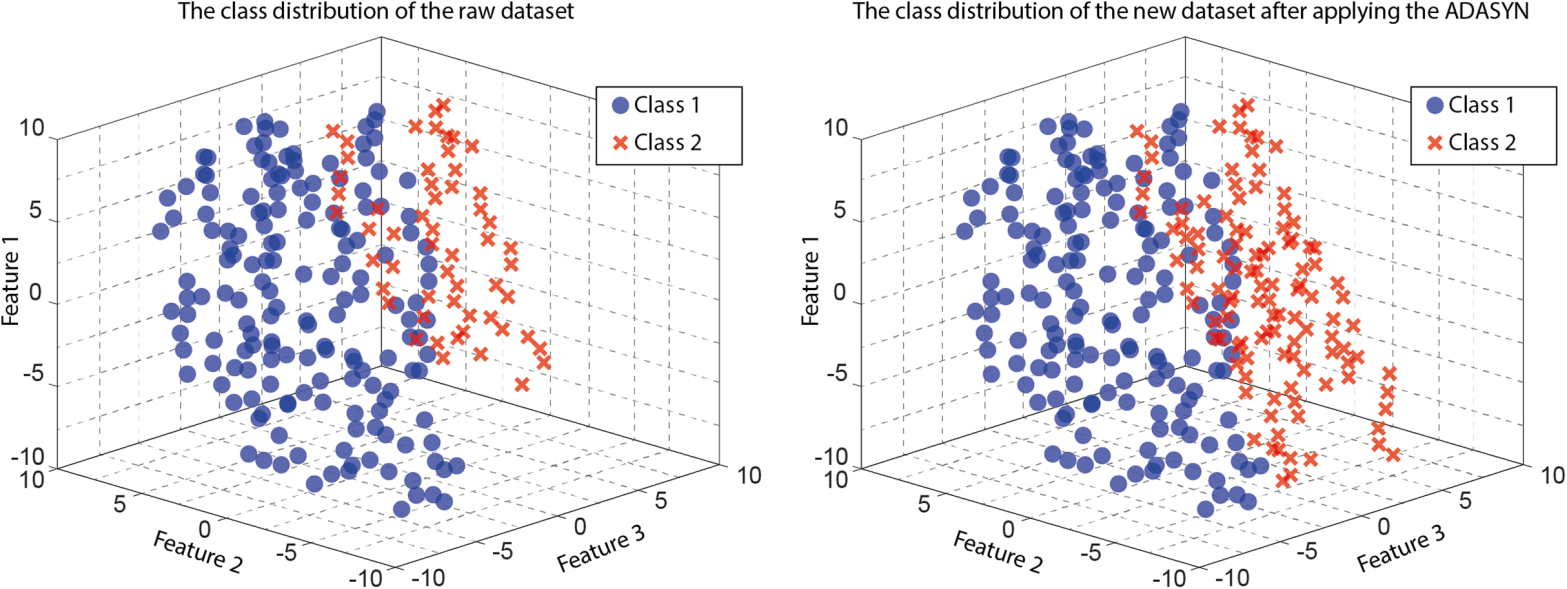
The working of ADASYN in a dataset. (A) presents the original dataset class distribution, and (B) shows the new dataset class distribution after applying ADASYN to the dataset (modified from https://medium.com/@ruinian/an-introduction-to-adasyn-with-code-1383a5ece7aa; http://glemaitre.github.io/imbalanced-learn/auto_examples/oversampling/plot_adasyn.html).

In this study, the ADASYN method (He et al., 2008) proposed by Haibo He et al. was used as a pretreatment before classification in the classification of multi-class EEG signals. The ADASYN method is an improved version of the SMOTE method (Fernandez et al., 2018). Its work is briefly as follows: It is run for two sets of data sets. For Minority and Majority classes, the data in the Minority class number approximates the data in the majority class. A good example is given in Fig. 7, showing the work of ADASYN (He et al., 2008; https://medium.com/@ruinian/an-introduction-to-adasyn-with-code-1383a5ece7aa; http://glemaitre.github.io/imbalanced-learn/auto_examples/over-sampling/plot_adasyn.html). The readers can refer to (He et al., 2008; https://medium.com/@ruinian/an-introduction-to-adasyn-with-code-1383a5ece7aa; http://glemaitre.github.io/imbalanced-learn/auto_examples/over-sampling/plot_adasyn.html) for more information about the working of the ADASYN algorithm.

### The classifier algorithm-Random Forests

The random forest classification algorithm is one of the popular machine learning models because it gives good results even without hyperparameter estimation and is applicable to both regression and classification problems.

**Figure 8:**
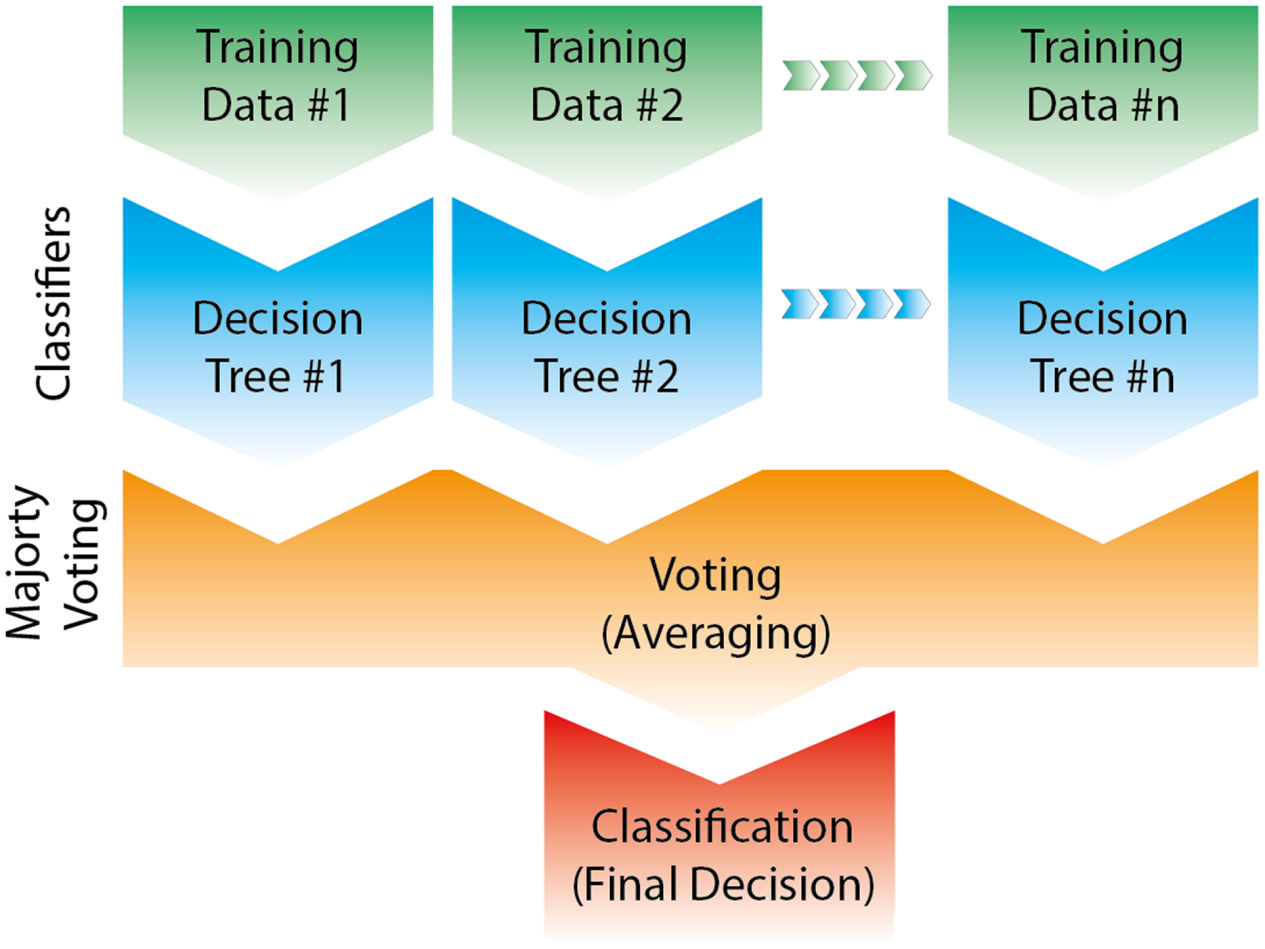
The working of Random Forests (RF) classifier algorithm in a two-dimension (modified from https://www.javatpoint.com/machine-learning-random-forest-algorithm).

Variance, in other words, overfitting, which is one of the biggest problems of decision trees, decreases since training is carried out on different datasets in the random forest model. In addition, the chance of being the outlier in sub-datasets created with the bootstrap method is reduced. Random Forests (RF) could be used for both classification and regression problems. Figure 8 shows the working of the Random Forests (RF) classifier algorithm in a two-dimension dataset (https://www.slideshare.net/0xdata/jan-vitek-distributedrandomforest522013; https://willsorenson.com/Everything_You_Need_to_Know_to_Use_Random_Forests.html). The readers can refer to these links (https://www.slideshare.net/0xdata/jan-vitek-distributedrandomforest522013; https://willsorenson.com/Everything_You_Need_to_Know_to_Use_Random_Forests.html; Breiman, 2001; Liaw, 2012; Daldal, 2020; Daldal, Cömert & Polat, 2020; Polat & Onur Koc, 2020; Arican & Polat, 2020; Ozdemir & Polat, 2020) for more information about the working of Random forests (RF).

## Results

In this study, four different approaches proposed by us for the first time in the literature are presented for the classification of multi-class EEG signals. In addition, the combination of one-against-all (OVA) and ADASYN (Adaptive synthetic sampling approach) algorithms were proposed by us and applied to the classification of EEG signals.

In the study, firstly, EEG signals are divided into four different bands by filtering methods: Beta, Alpha, Theta, and Delta. Then, the EEG signal and 125-time domain features were obtained from these four bands.

Four different models have been proposed and compared with each other. In the first method, the 125-TDF feature set was classified using the Random Forests (RF) classification algorithm, and the five-class EEG signals data set was classified with a classification accuracy of 71.90%.

**Figure 9:**
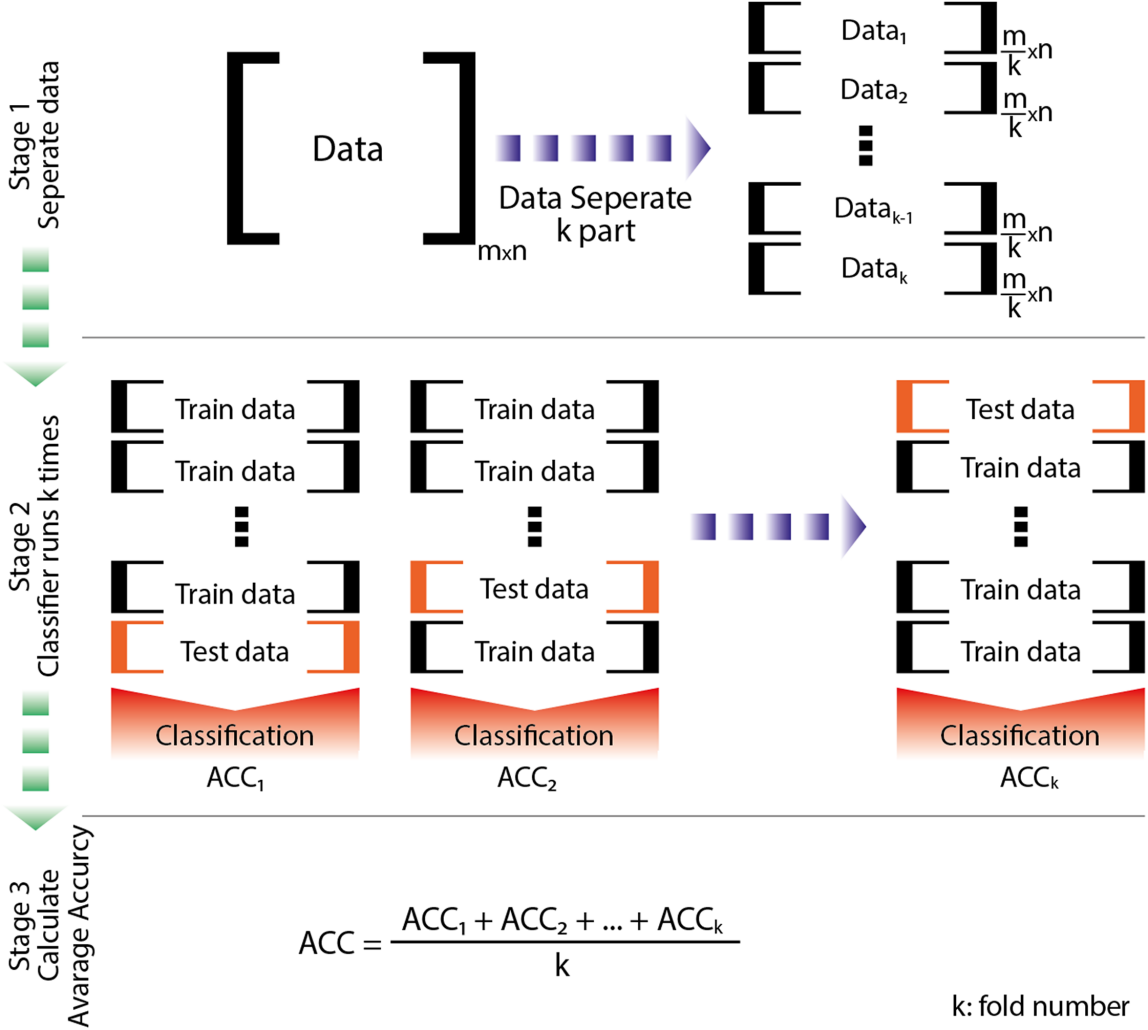
The working representation of the 5-fold cross-validation (modified from Zhang et al., 2019).

In the second approach, five different classes of EEG signals with a 125-TDF feature set were applied to one-against-one (OVO), and ten different pieces of data were obtained. Each piece of data is classified using a random forest (RF) classification algorithm. The majority voting scheme is used to combine ten different outputs. In training and testing of random forests (RF), the five-cross validation (5-CFV) method is used. The schematic representation of 5-CFV is shown in Fig. 9. Table 3 gives the classification accuracies of each piece after applied to OVO in the classification of multi-class EEG signals using a random forest classifier.

**Table 3:** The classification accuracies of each piece after applied to OVO in the Classification of multi-class EEG signals using Random Forests classifier with 5-FCV (the second approach).

The obtained piece after OVO	The obtained Classification accuracy (%)
Class 1-Class 2	96.90
Class 1-Class 3	98.30
Class 1-Class 4	98.20
Class 1-Class 5	99.80
Class 2-Class 3	64.10
Class 2-Class 4	95.60
Class 2-Class 5	90.70
Class 3-Class 4	94.50
Class 3-Class 5	91.40
Class 4-Class 5	81.30
The overall accuracy (average score)	91.08

In the third approach, in the 125-TDF dataset, five different data sets were obtained by applying the one-against-all (OVA) method to the five-class EEG signals dataset.5 different pieces of data have been obtained. Each piece of data is classified using a random forest classification algorithm. The majority voting scheme is used to combine five different outputs. ADASYN (adaptive synthetic sampling approach) has been used to increase the performance of the machine learning algorithm and to transform the unbalanced data set into balanced since the five pieces datasets obtained from OVA have an imbalanced data distribution. Table 4 shows the classification accuracies of each piece after applied to OVA in the classification of multi-class EEG signals using random forests classifier with and without ADASYN.

**Table 4:** The classification accuracies of each piece after applied to OVA in the classification of multi-class EEG signals using the Random Forests classifier with and without ADASYN using 5-FCV (the third and fourth approaches).

The obtained piece after OVO	The obtained Classification accuracy (%) Without ADASYN	The obtained Classification accuracy (%) With ADASYN
Class 1-others (the combination of class 2, class 3, class 4, and class 5)	98.10	98.40
Class 2-others (the combination of class 1, class 3, class 4, and class 5)	83.00	97.70
Class 3-others (the combination of class 1, class 2, class 4, and class 5)	84.60	88.50
Class 4-others (the combination of class 1, class 2, class 3, and class 5)	91.20	92.70
Class 5-others (the combination of class 1, class 2, class 3, and class 4)	88.10	91.50
The overall accuracy (average score)	89.00	91.72

## Discussion

In the classification of five class EEG signals, the results and comparison values of all the approaches used are given in Table 5. The results show that the best method is the fourth approach. In the fourth approach, the ADASYN method has increased the classification success. Also, the literature comparison is done in the classification of multi-class EEG signals and then given in Table 6.

**Table 5:** The classification accuracies obtained for five different approaches in the classification of multi-class EEG signals with 5-FCV.

The used method	The obtained Classification accuracy (%)
Alone Random Forests classifier with raw EEG signals	64.80
The first approach (the combination of 125-TDM features and Random Forests classifier)	71.90
The second approach (the combination of OVO and Random Forests classifier)	91.08
The third approach (the combination of OVA and Random Forests classifier)	89.00
The fourth approach (the combination of OVA, ADASYN and Random Forests classifier)	91.72

**Table 6:** The comparison of the other models and our work in the classification of multi-class EEG signals in the literature.

Work	The obtained Classification accuracy (%)
Tzallas, Tsipouras & Fotiadis (2009)	89
Liang, Wang & Chang (2010)	85.90
Alçіn Ömer et al. (2016)	96.40
Zahra et al. (2017)	87.20
Our work (2021)	91.72

## Conclusions

Brain signals are used safely in many areas. In particular, EEG signals are used in many areas such as the detection of neurological disorders, brain-computer interfaces, wheelchairs, and computer games. In this study, brain signals belonging to different situations were recorded, and four different approaches that were nice and applied for automatic detection of these EEG signals were proposed. A study has been carried out to be used in other areas from EEG signals, such as estimating epilepsy and detecting open and closed conditions.

In addition, the ADASYN (adaptive synthetic sampling approach) method and the one-against-all (OVA) methods were combined for the first time by us and applied to the classification problem of multi-class EEG signals. Also, we have proposed four different hybrid models to classify the multi-class EEG signals and then compared them with each other concerning the classification performance. In the fourth approach, the ADASYN method has increased the classification success in the classification of multi-class EEG signals using Random Forests classifier.

In the future, the proposed fourth approach could be used online in the detection of epilepsy based on the EEG signals. Also, the proposed systems could be applied to the BCIs (brain-computer interface).

## Additional Information and Declarations

### Data Availability

The following information was supplied regarding data availability: Source code is available at GitHub: https://github.com/kpolat14/multi_class_eeg_matlab_code. Data are available at GitHub: https://github.com/kpolat14/eeg-dataset/.
